# Laryngotracheoplasty in a low birth weight preterm newborn

**DOI:** 10.1590/S1808-86942012000100022

**Published:** 2015-10-20

**Authors:** Denise Manica, Cláudia Schweiger, Daniela Bruneli e Silva, Mariana Magnus Smith, Gabriel Kuhl

**Affiliations:** a Otorhinolaryngologist (Fellow of the Larynx, Clinic Hospital, Porto Alegre); b Otorhinolaryngologist (Hired medical doctor of the Clinic Hospital, Porto Alegre. Master's degree in pediatrics, Rio Grande do Sul Federal University); c Otorhinolaryngologist (Master's degree student, Rio Grande do Sul Federal University); d Otorhinolaryngologist (Master's degree in pediatrics, Rio Grande do Sul Federal University); e Otorhinolaryngologist (Professor of the Rio Grande do Sul Federal University)

**Keywords:** infant, newborn, intubation, laryngostenosis, larynx, tracheostomy

## INTRODUCTION

Stenosis is undoubtedly the most severe and most feared of airway injuries caused by endotracheal intubation. Retrospective studies have reported incidences of post-intubation subglottic stenosis in neonates ranging from 0.6 to 8.3%^1^, ^2^.

For laryngologists, stenosis of the larynx is one of the most challenging and complex conditions to treat. Management of subglottic stenosis in neonates remains controversial in spite of several therapeutic options.

## CASE REPORT

**Figure 1:**
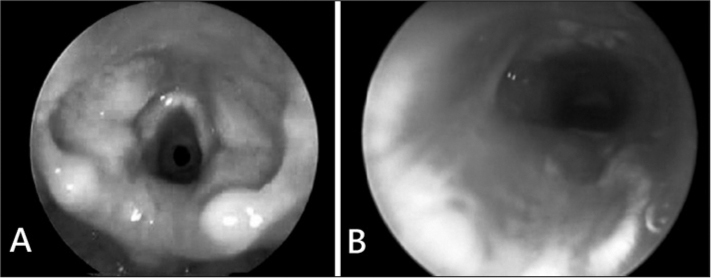
**A -** Direct laryngoscopy showing grade 2 (Myer-Cotton) subglottic stenosis. **B -** Direct laryngoscopy on the seventh postoperative day showing *in situ* grafts and good subglottic patency.

A female patient with a gestational age of 29 weeks and weight at birth of 1,035 g required endotracheal intubation at 10 hours of life due to sustained apnea. Endotracheal intubation was in place for three days. At 22 days of life, the patient was again intubated because of apnea. Eight days after removal of the endotracheal tube the patient presented tachypnea and a biphasic stridor. Direct laryngoscopy revealed a grade 2 (Myer-Cotton) subglottic stenosis^3^ (Figure 1-A).

Laryngotracheal reconstruction was the treatment of choice. At this point the patient was aged 1 month and 16 days of life, and weighed 1,670 g. Endotracheal intubation was done instead of a tracheostomy. The patient was placed in a position that extended her neck, the surgical site (anterior region of the neck and a thoracic site from which rib cartilage was taken) was anesthetized by infiltration (1% lidocaine and 1:100,000 adrenalin). The rib graft was harvested first to minimize contamination.

The site was the right submammary area next to the osteocartilaginous junction. Next, a horizontal neck incision was made and a subplatismal flap was prepared. The cricoid and thyroid cartilages and the trachea were identified. An anterior laryngofissure approach along the midline was done next. At this point the endotracheal tube was removed and placed below the stenosis; the posterior wall of cartilage was also opened.

The graft was prepared and placed first on the posterior wall with its perichondrium facing the internal portion of the airway and sutured with PDS 4.0. A nasotracheal tube was placed for modeling and graft material was placed over the anterior wall. A Penrose tube was placed and the wound was closed. The patient remained intubated postoperatively; direct laryngoscopy on the seventh postoperative day showed a good laryngeal lumen (Figure 1-B). The tube was removed and the patient had no respiratory dysfunction. The patient was discharged 20 days after the procedure; at this point she weighed 2,090 g.

Revision direct laryngoscopies on the 1^st^ and 3^rd^ months postoperatively showed that there was no stenosis. The patient currently is aged 3 years and is symptom-free.

## DISCUSSION

We reported the case of a neonate weighing less that 2 Kg that presented subglottic stenosis; from our perspective, the choice of treatment for a safe airway were l laryngotracheal reconstruction or tracheostomy until the child achieved more weight and age.

Until the 1950s, tracheostomy was the only treatment described for subglottic stenosis. Cotton argued that tracheostomy was the procedure for keeping the airways safe in neonates with subglottic stenosis until they achieved more weight and grew to withstand reconstructive surgery^4^. However, tracheostomy in neonates is risky because the cannula gauge is very small, and there is a high possibility of obstruction due to secretions. Furthermore, because of poor social and economic status among the population seen at public hospitals in Brazil, management of children with tracheostomy at home may be very difficult.

There are few published papers on the surgical management of subglottic stenosis in neonates and lactating infants.

Agrawal et al reported their experience with laryngotracheal reconstruction in 37 children aged from 3 to 66 months; the mean stenosis grade in these cases was 2.5. The authors did not report the mean weight of the subjects. Their reported rate of surgical success was 89%^5^.

Triglia et al reported their experience of 141 surgeries to treat subglottic stenosis in infants; the procedures included the anterior cricoid split procedure, laser surgery, laryngotracheal reconstruction, and laryngotracheal resection. Their sample had no children under 2 Kg. Their general success rate was 94%^6^.

We chose to carry out laryngotracheal reconstruction in the case, even though we found no published reference on children with such a low weight. This choice was made because of the risks of tracheostomy in this patient.

## FINAL COMMENTS

We described the case of a premature neonate with post-intubation subglottic stenosis that underwent laryngotracheal reconstruction at the age of 1 month and 16 days and weighing 1,670 g.

The decision to carry out tracheostomy or laryngotracheal reconstruction in neonates with subglottic stenosis should be made in the context of a multidisciplinary team. The medical status, risk of surgery, chances of success of the procedure, and difficulty in managing a tracheostomy at home should be considered. The age and weight of neonates are debatable issues, and are not decisive for choosing the best treatment for correcting subglottic stenosis.
